# Tumor Microenvironment Role in Pancreatic Cancer Stem Cells

**DOI:** 10.3390/cells12121560

**Published:** 2023-06-06

**Authors:** Aaron Galindo-Vega, Vilma Maldonado-Lagunas, Irma B. Mitre-Aguilar, Jorge Melendez-Zajgla

**Affiliations:** 1 Functional Genomics Laboratory, Instituto Nacional de Medicina Genómica, Mexico City 04710, Mexico; aarongave4648@gmail.com; 2 Epigenetics Laboratory, Instituto Nacional de Medicina Genómica, Mexico City 04710, Mexico; vmaldonado@inmegen.gob.mx; 3 Biochemistry Unit, Instituto Nacional de Ciencias Medicas y Nutricion Salvador Zubiran, Mexico City 14080, Mexico; irma.mitrea@incmnsz.mx

**Keywords:** cancer stem cells, tumor microenvironment (TME), PDAC

## Abstract

Pancreatic ductal adenocarcinoma (PDAC) is a highly lethal malignancy with a majority of patients presenting with unresectable or metastatic disease, resulting in a poor 5-year survival rate. This, in turn, is due to a highly complex tumor microenvironment and the presence of cancer stem cells, both of which induce therapy resistance and tumor relapse. Therefore, understanding and targeting the tumor microenvironment and cancer stem cells may be key strategies for designing effective PDAC therapies. In the present review, we summarized recent advances in the role of tumor microenvironment in pancreatic neoplastic progression.

## 1. Introduction

Pancreatic cancer (PC), despite being the 13th most common cancer, has remained the most lethal malignancy globally [1,2]. This is due to its low 5-year survival rate of only 1–9% [1,3,4,5,6], which can be attributed to late diagnosis-with 95% of cases detected in highly advanced stages where chemotherapy and radiotherapy options are limited [7,8]. In addition, PC has a high cellular heterogeneity and a strong desmoplastic reaction, which decrease chemotherapy effectiveness. Pancreatic exocrine cells produce enzymes involved in the metabolism of proteins, carbohydrates, and lipids, represent 90% of the organ mass so, and are responsible for 90–95% of pancreatic malignancies originating in this tissue. Of these, 90–95% correspond to the most common histologic and aggressive subtype of PC, pancreatic ductal adenocarcinoma (PDAC) [9,10]. Intratumoral heterogeneity in these tumors is profound since it has been shown that up to 1000 genes are differentially expressed in cancer cells between the tumor center and its periphery [11]. In addition, there is also a deep heterogeneity among PC of different patients, which adds to the complexity of the disease [12]. The high cellular heterogeneity is due in part to the presence of cancer stem (or tumor-initiating) cells, which have been extensively reported in these tumors.

This review aims to address the pancreatic cancer stem cells (PCSCs) theory from the point of view of TME and the different possible therapeutic approaches against them.

## 2. Clonal Evolution of Tumoral Populations

**Figure 1:**
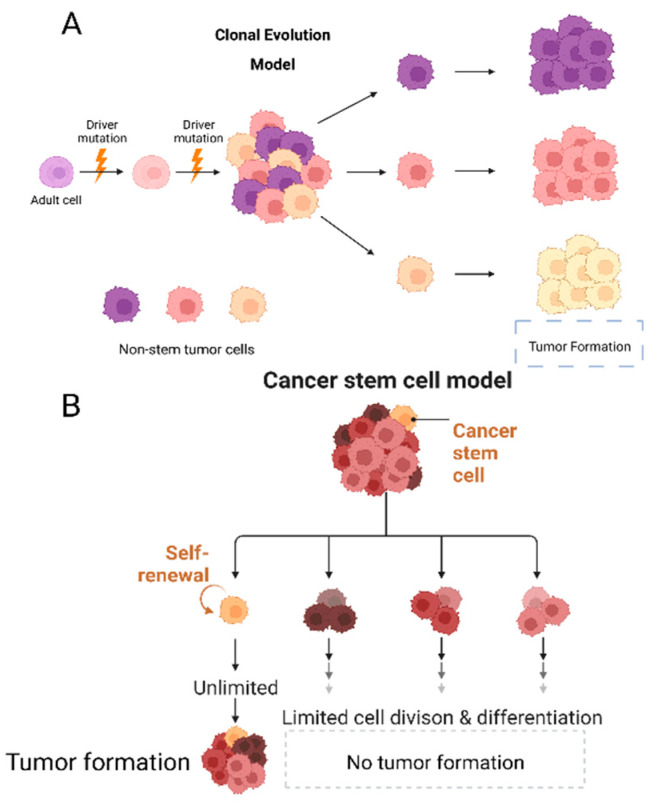
Cancer heterogeneity can be explained by two different models. The first model is the clonal evolution model (**A**), where stochastic mutations give rise to the tumor, and every tumor cell has the potential to generate a new tumor. The second model is the cancer stem cell model (**B**), in which tumors arise from mutations in stem cells or progenitor stem-like cells, and only the cancer stem cells can sustain and form new tumors.

There are two prevailing theories seeking to explain cancer. The first, which is the clonal evolution theory, is rooted in evolutionary biology as proposed by Peter Nowell (1976) (see Figure 1A). This theory suggests that random driver mutations take place in tumor cells, which are selectively clonally chosen to provide a growth advantage compared to other cells in the tumor and adjacent normal cells. According to this theory, any cell within the tumor can initiate a new tumor or uphold tumor growth, implying that all cells possess the same tumorigenic potential [13]. The second theory, which is not mutually exclusive, postulates the presence of cancer stem cells.

## 3. Cancer Stem Cells (CSCs)

Cancer stem cells, also known as CSCs, exhibit similar traits to their normal tissue stem cell counterparts. These include the ability for self-renewal and asymmetric division, as well as characteristics such as radio and chemotherapy resistance, immune evasion, and the capability for infinite proliferation [14,15,16]. This last feature also contributes to cancer progression, since it facilitates the acquisition of mutations that become the target of evolution to a more aggressive tumor (Figure 1B) [3,17]. Cancer stem cells (CSCs) exhibit several mechanisms to resist chemotherapy and radiotherapy. While conventional therapies aim to destroy rapidly dividing cells, CSCs have the ability to enter a quiescent state (G0 phase) and lay dormant until they are triggered to re-enter a proliferative state under favorable conditions or when required [18,19]. Furthermore, in the case of cancer stem cells, there is an increased number of ATP-binding cassette (ABC) transporters (such as ABCB1, ABCC1, and ABCG2) whose primary function is to expel drugs or foreign substances from the cell [20]. CSCs present intrinsic activated signaling mechanisms that help them avoid apoptosis, such as PI3K, Wnt/β-catenin, and NOTCH pathway [21]. The tumor microenvironment is another crucial aspect of treatment resistance, as it plays a significant role in therapy failures and cancer relapse. The cellular and acellular components of the microenvironment can decrease the efficacy of chemotherapy and radiotherapy, emphasizing their importance in determining treatment outcomes [22].

CSCs were first described in 1994 when Lapidot et al. studied acute myeloid leukemia xenotransplants in immunocompromised mice. These authors noticed a group of cells that maintained the progenitor leukemic cell pool and used flow cytometry to isolate them and named them leukemic initiating cells or leukemic stem cells [23].

These authors used the surface protein marker CD34, which is expressed in normal hematopoietic progenitor and pluripotent stem cells of the bone marrow, and the CD38 lineage marker, to ensure an immature cell lineage. Isolated CD34^+^CD38^+^ cells implanted into immunocompromised SCID mice developed the disease, whereas CD34^+^CD38^−^ cells did not [23].

Epithelial-to-mesenchymal (EMT) transition occurs naturally in an organism’s development, particularly during gastrulation and neural cord formation, as well as in wound healing. However, EMT has also been linked to carcinogenesis, specifically in tumor invasion, migration, and metastasis. This cellular mechanism causes the loss of cell-cell tight junctions and epithelial molecular markers in epithelial tumor cells, allowing them to spread into the bloodstream and colonize distant tissues and organs. Furthermore, EMT has been associated with cancer stem cells (CSCs), as cells with EMT traits exhibit features of stemness markers such as SOX2, KLF4, and OCT4, as well as higher expression of CSC surface markers CD44, CD24, EpCAM, or CD133 in various cancer types, including pancreatic ductal adenocarcinoma (PDAC) [24]. 

The origin of cancer stem cells (CSCs) remains a topic of controversy in the scientific community [25,26]. It has been established that this key subpopulation involved in tumor progression and origin may arise from either normal stem cells due to their intrinsic ability to divide and proliferate indefinitely. Thus, accumulating more driver mutations than non-stem cells that aid in oncogenic processes. Alternatively, there is also evidence that CSCs may arise from early progenitors or differentiated normal/tumor cells that acquire stem-like capacities cells [27,28,29]. The origin of CSCs is, however, dependent on the tissue type or tumor. Studies have shown that in gastrointestinal cancers, CSCs arise from normal or healthy stem cells and not from progenitor or differentiated cells [30]. Interestingly, research conducted in acute myeloid leukemia (AML) has shown that primitive normal cells, and not progenitor cells, are the cell-of-origin in this malignancy [31]. More surprisingly, in a recent in vitro experiment, mesenchymal stem cells (MSCs) have also been shown to act as the origin of cancer stem cells through spontaneous transformation [32].

## 4. Pancreatic Cancer Stem Cells

**Figure 2:**
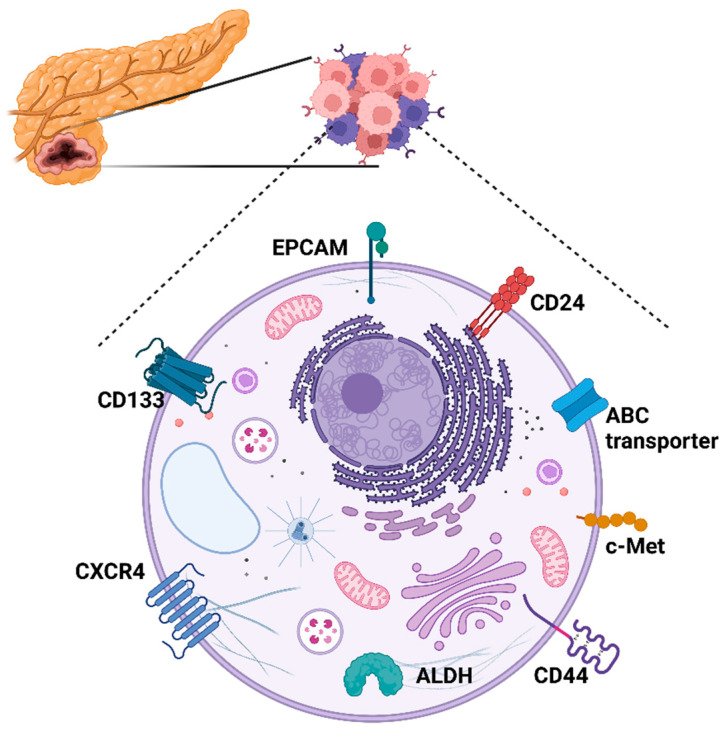
Representative scheme of the different markers found in pancreatic CSCs. ATP-binding cassette (ABC); Epithelial cell adhesion molecule (EpCAM); Aldehyde dehydrogenase 1 (ALDH); C-X-C chemokine receptor 4 (CXCR4). Different combinations of cell surface markers provide the possibility of isolating clones with higher or lower aggressiveness.

CSC were first isolated in solid tumors from breast cancer samples [33]. Using similar membrane markers, CSC have been found in almost all solid tumors. In 2007 Li et al. analyzed primary tumors and metastatic lesions from PC patients using the cell surface markers CD44+, CD24+, and EPCAM+, alone or in different combinations (Figure 2). These authors found that isolated cells that were positive for each of the individual markers (for example, CD44+) had increased tumorigenic potential, as compared to their negative counterparts, following injection into NOD/SCID mice. Among all the different possible combinations, the more aggressive and tumorigenic potential was exhibited by CD44+CD24+EPCAM+ cells. Mice implanted with as few as 100 cells developed pancreatic tumors whereas implanting hundreds or thousands of negative cells did not. Interestingly, these cell populations represented only 0.2–0.8% of the total population. Additionally, they observed that the tumors formed in SCID mice showed the same histological features as the primary tumors from which they were isolated, thus generating every different cellular subtype found in a tumor. The conclusion of this work was the existence of a subpopulation with cancer stem cell properties and increased tumorigenic potential [34].

Several more papers aimed to analyze and characterize this small cell subpopulation have since been published. For example, Hermann et al. identified a PDAC stem population in patients’ primary tumor samples with metastatic abilities based on the premise that if this cell population can initiate and maintain tumors, they could also have the ability to migrate and metastasize [4]. To address this, they isolated CD133+ cells through flow cytometry, since this marker is expressed by both normal and malignant stem cells from various embryonal lineages. Their results showed that pancreatic cancer stem CD133+ cell population exhibits a greater tumorigenic potential than CD133− cells or non-sorted cells since as few as 500 cells CD133+ were able to initiate a tumor implanted in nude mice. In contrast, 1 million CD133− cells were not able to do this. In addition, the CD133+ population showed resistance to the most common chemotherapeutic drugs used in PDAC, such as gemcitabine. Interestingly, the authors observed a partial overlap among their subpopulations and Li et al. subpopulations [4].

In the years following the discovery of the pancreatic ductal adenocarcinoma (PDAC) stem cell marker CD44, Li et al. identified another key stem tumor marker: c-Met. This protein is part of the receptor tyrosine kinase family and is involved in several significant tumoral processes. In their work, the authors analyzed the presence of other cancer stem cell markers, including CD44+/CD24+/EPCAM+ and CD133+. An overlap in stem cells defined by each of these markers was observed. Interestingly, the most tumorigenic subpopulation was found to be c-met/high/CD44+, with as few as 50 cells enough to develop pancreatic cancer in NOD/SCID mice, where the percentage of c-met and c-met/high cells ranged between 2–16%. Significantly, when mice were treated with gemcitabine–the drug commonly used to treat PDAC–cancer stem cell population increased by up to 50%, potentially explaining why some types of cancers relapse after radio or chemotherapy. To determine if c-Met played a role in sustaining cancer stem cell population, the study used XL184, a potent small kinase inhibitor of c-Met. Combination treatment with XL184 and gemcitabine led to a significant reduction in tumor size, inhibition of metastasis up to 32 days after treatment was stopped, and a suppressed spheroid formation. This suggests that targeting both the tumor bulk and cancer stem cells through drug combinations is essential to preventing tumor relapse and improving the prognosis of PDAC and other types of cancer [35]. 

There are numerous methods used to identify pancreatic cancer stem cells (CSCs), including their ability to form spheres from pancreatic cancer cell lines and metastatic foci under non-adherent culture conditions. Furthermore, pancreatic CSCs exhibit higher expression levels of pancreatic CSC markers and the ability to efflux chemotherapy drugs in these spheres. This is proven by the side population (SP) that can be identified due to their incapacity to be stained with DNA dyes, thus excluding them from the major population (MP). The SP also exhibits features of stem-like cells, as evidenced by their ability to form larger tumors than the MP. Additionally, recent research has shown that the SP can exclude some chemotherapeutic drugs, such as gemcitabine [36].

## 5. Pancreatic Cancer Stem Cells Origin

It has been widely suggested that cancer stem cells (CSCs) may arise from normal tissue stem cells due to their long lifespan and increased cell division rates for the purpose of maintaining tissue homeostasis. This proclivity towards cell division increases the likelihood of acquiring mutations that provide a competitive advantage over surrounding cells, leading to the development of cancer. Several studies have shown that the main cell lineages of the pancreas, which express the transcription factor pancreatic and duodenal homeobox 1 (*Pdx1*), arise from the same progenitor cells. These progenitor cells have the potential to give rise to all the cells that comprise pancreatic tissue. However, these progenitor cells are no longer present later on, and the regeneration of the pancreas does not depend on them [37,38,39],. Currently, there is no conclusive evidence for the existence of pathologically relevant ductal stem cells in the adult pancreas. Currently, there are only a few reports of ductal stem cells in engineered mice [40] and single cell-RNA-seq studies [41] that have shown the presence of stem-like or progenitor-like cells subpopulation in cultured on in vitro conditions that are not physiologically accurate. In addition, a pancreatic ductal gland (PDG) compartment, which is an epithelial zone where the pancreatic stem cells reside, has been found. Cells within this compartment could be involved in pancreatic ductal epithelial regeneration from cells expressing core factors related to embryonic stem cells [42]. Nevertheless, the PDG compartment is not involved in the regeneration after an inflammatory injury of the terminal ducts in the ductal epithelium, nor of the acinar cells. Therefore, it has been shown that, in case of injury, the pancreas presents an alternative pathway to repair tissue damage. This mechanism makes use of cells that have some degree of plasticity in the acinar (the most plastic cell type) and ductal cell compartment. This phenomenon is known as the acinar to ductal metaplasia (ADM). In this process, the acinar cells (the pancreatic cells with the highest plasticity) undergo de-differentiation to an embryonic-like state and later re-differentiate to a ductal cell or any other necessary cell type. This event has been proposed as the origin of PDAC, as supported by studies in transgenic mice and 3D culture of human pancreatic cells, in which, after de-differentiation, mutated *KRAS* hyperactivity and inflammatory factors make this step irreversible. These cells then give rise to precancerous pancreatic intraepithelial neoplasia (PanIN1 or PanIN2). This is not enough for the appearance of cancer thought, but the clonal advantage in these cells makes them prone to additional mutational events, such as the activation of the epidermal growth factor receptor (*EGFR*), NOTCH signaling or the recruitment of macrophages and pro-inflammatory factors. Interestingly, there is evidence that low-grade PanIN lesions cells positive for DCKL1 show similar properties to CSCs [43,44]. It has been observed that cancer stem cells (CSCs) can undergo a process called epithelial to mesenchymal transition (EMT) at some point during their development, which gives them the ability to metastasize-a characteristic hallmark of CSCs. Research suggests that acinar cells are the cell of origin for pancreatic ductal adenocarcinoma (PDAC), with EMT as the initial mechanism. The recent study by Peng et al. supports this theory, as they performed single-cell sequencing on both primary tumor samples and normal adjacent tissue. By analyzing the progression of cells using a trajectory analysis known as pseudo-time, the authors found that the disease initiated from acinar cells and cells expressing low levels of muc1, which gradually progressed towards cells expressing high levels of muc1 and malignant ductal type 2 cells [45]. While acinar cells are considered a possible cell of origin for pancreatic ductal adenocarcinoma (PDAC), ductal cells are also a potential source of the disease via preinvasive lesions known as intraductal papillary mucinous neoplasms (IPMN). Ductal cells can undergo a dedifferentiation process characterized by the expression of progenitor markers such as pdx1 and hnf4a, which later leads to the development of metastatic PDAC. it is important to note, however, that these instances are much less common than those resulting from pancreatic intraepithelial neoplasia (PanIN) lesions [46].

## 6. Tumor Microenvironment (TME)

**Figure 3:**
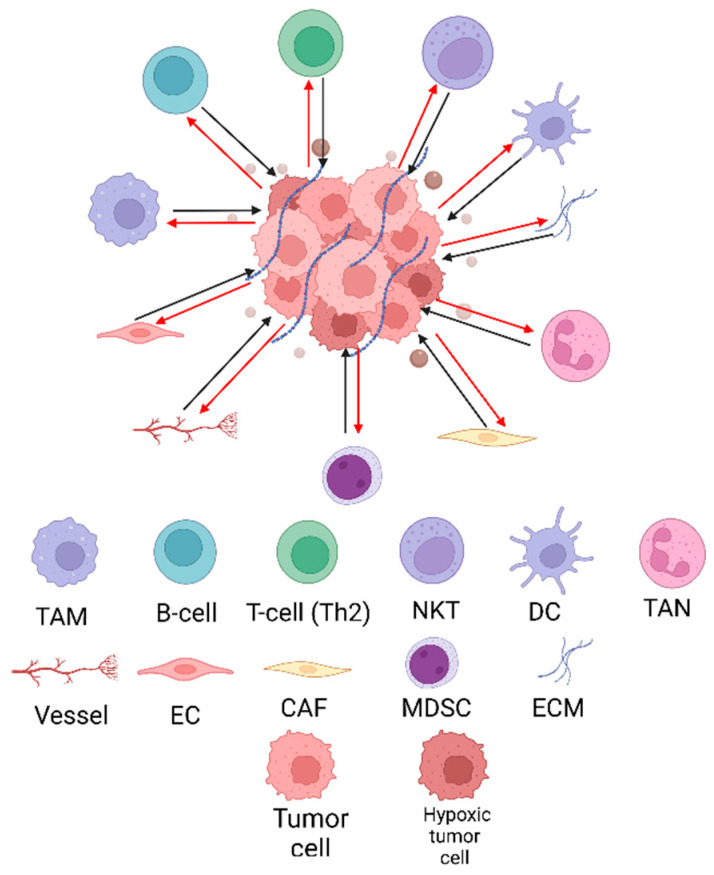
Schematic representation of the different cellular and acellular components of the TME. The interplay between the tumor and surrounding environment, known as the tumor microenvironment (TME), involves a myriad of cellular and acellular components, constantly interacting and influencing each other. The crosstalk between malignant and non-malignant cells plays a significant role in cancer progression and treatment response.

There is a lot of cumulative evidence about the importance of studying the tumor microenvironment (TME), a term first used by Virchow in 1889 when proposing the relationship between inflammation and cancer. The TME is formed by acellular and cellular compartments. The former corresponds to different molecules such as cytokines, chemokines, and vesicles, as well as extracellular matrix (ECM) components. The latter refers to malignant and non-malignant cells, such as tumor-associated macrophages (TAMs), B-cells and T-cells, natural killer cells (NK) and natural killer T cells (NKT), dendritic cells (DC), tumor-associated neutrophils (TANs), myeloid-derived suppressor cells (MDSC), endothelial cells (EC), and probably the most abundant of all, cancer-associated fibroblast (CAFs) (Figure 3) [47,48,49]. The various components of the tumor microenvironment collaborate to evade immune detection and minimize the efficacy of drug therapies. 

**Figure 4:**
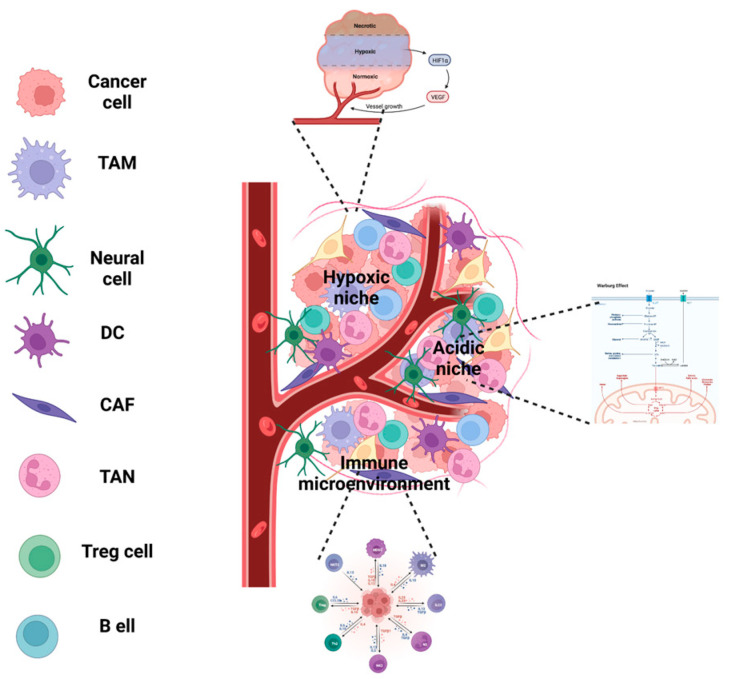
Within the TME, there are various specialized microenvironments, including the hypoxic, acid, immune, and metabolic niches. These niches continuously interact and communicate with cellular and acellular components, influencing the efficacy of therapies, as well as the progression, evolution, and prognosis of various cancer types.

TME plays a crucial role in cancer development and progression. It involves continuous communication between malignant and non-malignant cells and contributes to various aspects of cancer, including tumor formation and maintenance, metastasis, immune evasion, and resistance to chemotherapy and radiotherapy. The acquisition and maintenance of cancer hallmarks largely depend on the TME. Several studies have shown that cancer cells manipulate non-malignant cells by releasing molecules and vesicles to mediate the release of growth factors and other molecules that stimulate cancer cells to undergo metastasis or attract immune cells. The interactions between cellular and acellular components are also crucial to the generation of tumor heterogeneity and clonal evolution [50]. These elements contribute to the development of hallmark features within the tumor microenvironment, which forms and regulate specialized niches. Such specialized niches have been implicated in therapy resistance and immune evasion. At least six distinct niches within the tumor microenvironment have been reported, namely: the hypoxic, acidic, mechanical, innervated, metabolic, and immune niches (Figure 4) [51]. 

Several researchers suggest that non-malignant cells within the tumor microenvironment (TME) may serve as promising therapeutic targets, as they are genetically more stable and exhibit less resistance to chemotherapy and radiotherapy. Alternatively, rather than targeting the cellular compartment, targeting acellular components within the TME, such as hyaluronic acid, extracellular matrix, exosomes, cell-free DNA and apoptotic bodies could be more successful, as these elements are even more stable. Nevertheless, it is crucial to take into consideration the differences between the tumoral and normal microenvironment present elsewhere in the body to prevent potential cytotoxicity [50,52,53,54].

### 6.1. Hypoxic Niche

The high proliferation rate and inadequate vascularization of tumors create a state of oxygen deprivation, leading to the formation of hypoxic niches. Studies indicate that hypoxia-inducible factor 1 (HIF-1) and its signaling pathway are responsible for the transition from normoxia to hypoxia [55]. This response triggers several functions, including the regulation of stemness through transcription factors (such as SOX2, KLF4, NANOG, MYC, and OCT4) that promote cancer cell survival and sometimes correlate with poor prognosis and tumor progression [56,57]. Additionally, HIF-1 stimulates the expression of pro-angiogenic factors, with vascular endothelial growth factor (VEGF) [58] being the most relevant. Moreover, HIF-1 is involved in the epithelial-to-mesenchymal transition by activating transcription factors like Twist, Snail, and ZEB1 [21,59]. These findings suggest that the hypoxic niche may be a viable therapeutic target.

### 6.2. Acidic Niche

Healthy cells derive energy through oxidative phosphorylation while glycolysis is typically inhibited under normal oxygen levels. However, cancer cells exhibit metabolic reprogramming and show a preference towards glycolytic metabolism, even in the presence of oxygen, which is known as the Warburg effect or aerobic glycolysis. Although this pathway is less efficient than oxidative phosphorylation and generates only 2 ATP molecules, it is faster and can produce larger quantities of cellular energy [60,61]. A drawback of glycolytic metabolism is the excessive secretion of lactate, which can cause a drop in pH and acidify the tumor microenvironment. Initially, this is detrimental to cancer cells, as it can promote apoptosis, but subsequent adaptation to the acidic environment can provide tumorous cells with greater advantages over adjacent healthy cells [62]. For one, lactate has been seen to shift macrophages’ polarization towards a pro-inflammatory and pro-tumorigenic state [63,64]. It is also associated with the hypoxic niche and is known to be involved in tumor cell survival in a low-oxygen environment [65]. Furthermore, lactate plays a role in the attraction and maintenance of regulatory T-cells, thereby contributing to the immunosuppressive environment that enables the evasion of immune surveillance [66].

### 6.3. Mechanical Microenvironment

This highly specialized niche recently described [67,68], is composed of several components, both intracellular (such as vimentin, actin, and neurofilaments) and extracellular (such as collagen) [69]. This niche is intimately linked to the EMT as well as to the secretion of matrix metalloproteinases (MMPs) by stromal cells (CAFs), which are involved in the remodeling of the extracellular matrix (ECM). As expected by this, the niche has the ability to induce metastasis [70,71]. 

### 6.4. Innervated Niche

Recently, it has been shown that the nervous system influences the formation, maintenance, and progression of cancer. The release of neurotransmitters or neuropeptides stimulates the growth and progression of the tumor, either by pre-existing nerves or neo-neural networks derived from surrounding cells [72,73]. The neural network within tumors can also help tumor cells to follow a route to spread to other organs and tissues [74,75]. Similarly, it has been described that neural progenitors can migrate from the brain to the site of the tumor in order to form neurons, either de novo or through recruitment [76,77]. This highly specialized microenvironment has been described in different cancer types such as breast, pancreas, prostate, colon, head and neck, and ovarian [78,79]. Interestingly, the innervated niche present in brain tumors such as astrocytoma, schwannoma, glioma, or brain metastases is rather different than the innervated niche present in tumors from other organs [80,81,82]. Tumor innervation has been associated with more aggressive tumors as well as poor prognoses in terms of therapy outcomes, as tumor innervation promotes more complex communication among tumor cells, stromal cells, endothelial cells, immune cells, and the extracellular matrix, leading to resistance or therapy failure [72,74,83]. This makes the innervated niche a potential therapeutic target for anti-neurotrophic treatments. 

### 6.5. Metabolic Microenvironment

The metabolic microenvironment refers to the nutrient availability within the TME and how tumor cells exploit these nutrients to their metabolic advantage. One of the most important components is lactate metabolism, which we previously described in the hypoxic and acidic niches. Tumor cells use aerobic glycolysis to produce energy even under normal oxygen conditions, resulting in large amounts of lactate that acidify the environment and modulate the function of several immune system cells. However, lactate metabolism is not the only metabolic change that tumor cells undergo. Reactive oxygen species (ROS) are present in the tumor microenvironment and can be produced by both cancer and stromal cells, causing damage to cell integrity [84]. However, tumor cells are able to adapt in order to achieve a balance with ROS, a phenomenon known as ROS addiction [85]. ROS are also able to recruit and regulate immune cells, such as MDSC, TAMs, and regulatory T cells [86,87,88].

### 6.6. Exosomes

Extracellular vesicles (EVs) are small membrane-bound particles secreted by cells that can carry and transport a variety of biological molecules [89,90]. Initially, it was thought that these vesicles were only formed as part of cellular waste products or due to cellular damage [91]. However, today we know that these vesicles have much more complex functions, as they can transport very important information inside or on their surface, such as signaling molecules, ECM proteins, nucleic acids, transcription factors, enzymes, and growth factors, and have been postulated as a way of cellular communication [92,93,94]. There are several types of EVs, such as microparticles, exosomes, and apoptotic bodies, among others [95,96]. Extracellular vesicles (EVs) can be formed through two mechanisms: direct shedding from the cell membrane or exocytosis [91,97]. They are found in both prokaryotic and eukaryotic cells [98], highlighting the evolutionary conservation of this cellular communication system. While EVs have essential implications in physiological contexts, they have a crucial role in disease states, particularly cancer [99,100]. 

Exosomes are double-membrane vesicles with a size ranging from 50 to 150 nm (average diameter of 100 nm) [101,102]. Exosomes play a crucial role in promoting tumor progression through various mechanisms. It has been demonstrated that cells present in tumors release a higher number of exosomes compared to those under physiological conditions [103]. Furthermore, exosome-mediated communication has been observed between cancer-associated fibroblasts (CAFs), macrophages, and tumor cells in promoting the hypoxic niche and autophagy, thus aiding tumor progression under certain conditions [104,105]. One such condition is an acidic pH environment, where in some types of cancer such as melanoma, higher exosome production has been observed under acidic pH conditions than under normal pH conditions [106]. Similarly, under hypoxic conditions, a higher accumulation of mir-123 was observed in exosomes of lung cancer tumors, which promotes the expression of HIF-1α and enhances angiogenesis, leading to tumor progression and maintenance [107]. Other cancer types have also shown increased production and secretion of exosomes under hypoxic conditions [108,109,110]. Additionally, exosomes have been implicated in macrophage polarization, which transforms anti-tumoral M1 macrophages towards a pro-tumoral M2 phenotype that can suppress the immune response [111]. For these reasons, exosomes have been proposed as novel therapeutic targets. For example, in a recent study conducted in pancreatic cancer, exosomes were loaded with a Galectin-9 siRNA which would prevent macrophages from polarizing towards a pro-tumoral M2 phenotype [112]. Exosome analysis could be also used as a diagnostic tool since they can be found in the bloodstream in the early stages of the disease [113].

## 7. TME in PDAC

**Figure 5:**
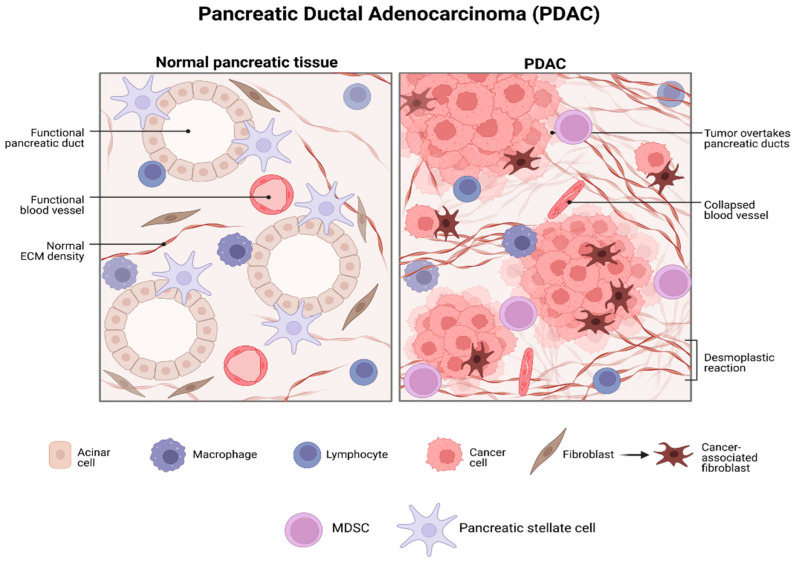
PDAC TME. Schematic representation of the microenvironment in the normal pancreas (**left**) with a regular tissue architecture and the tumor microenvironment in PDAC (**right**) in which we can observe high desmoplastic zones, unrecognizable pancreatic architecture, immune infiltration, and a large number of stromal cells, increased amounts of ECM and the lack of organized blood vessels (which causes hypoxic conditions).

The TME in PDAC is characterized by a dense stroma and increased desmoplasia. The source of this is the CAFs, which make up approximately 90% of the tumor mass. The main source of CAFs is the pancreatic stellate cells (PSCs), which become activated after an injury or chronic inflammation. CAFs release large amounts of ECM, including hyaluronans and collagen. This contributes to the formation of a solid and dense physical and biological barrier in the PDAC microenvironment, preventing the infiltration and proper functioning of immune cells such as B and T cells. This stroma not only makes the delivery of chemotherapeutic drugs more difficult, but it also causes an increase in interstitial pressure leading to hypovascularization and hypoxia, another crucial hallmark of the PDAC microenvironment (Figure 5). In turn, hypoxia leads to the activation of PSCs, causing positive feedback and more desmoplasia that favor disease progression. The tumor microenvironment (TME) is highly innervated in pancreatic ductal adenocarcinoma (PDAC). A recent study conducted on 132 PDAC patients demonstrated the presence of neural cells within the tumors. This is due to the fact that tumoral cells release neurotrophins to facilitate the neural infiltration and growth of the tumor [114,115,116]. These neural cells form a feedback loop by producing molecules such as catecholamines that bind to β-adrenergic receptors located on the surface of the cancer cells. This process promotes tumorigenesis and contributes to the progression of PDAC. The neural cells present in the tumor microenvironment form a feedback loop, producing molecules such as catecholamines that bind to β-adrenergic receptors on the surface of cancer cells. This process ultimately promotes tumorigenesis [117,118]. The most abundant immune cells in TME in PDAC are tumor-associated macrophages (TAMs), myeloid-derived suppressor cells (MDSCs), and T-regulatory cells. The M2-like phenotype macrophages, T-reg cells, and MDSCs block or suppress the infiltration of CD4+ and CD8+ T cells in the PDAC TME, creating a highly immunosuppressive environment [119,120,121]. Pancreatic ductal adenocarcinoma (PDAC) is recognized as having poor immunogenicity and an immunosuppressive microenvironment. While checkpoint inhibition therapy has demonstrated success in treating other cancers, its effectiveness in PDAC patients has been limited thus far.

Over the past decade, Single-Cell RNA Sequencing (sc-RNAseq) has undergone significant modifications to improve its depth and range, resulting in numerous achievements in various fields including cancer research. Through the utilization of sc-RNAseq, researchers can examine the different levels of complexity within this disease. sc-RNAseq facilitates the study of transcriptomics, as well as genomics, through methods such as sc-DNAseq, and it enables the evaluation of post-transcriptional modifications in chromatin through sc-ATACseq. The use of such techniques has become an indispensable tool for investigating the intricacies of cancer at a single-cell level [122,123]. Recently, PDAC’s TME characterization has gained traction due to the availability of these technologies, including single-cell RNA sequencing (sc-RNASeq), spatial transcriptomics, and multimodal genomic-proteomic approaches, supporting the initial discoveries that showed the presence of distinct stromal components, such as structural vascularized, activated, inflammatory and immune [124,125]. For example, a multimodal approach employing cytometry time-of-flight (CyTOF) immune phenotyping, scRNA-Seq, and multiplex fluorescent immunohistochemistry (mfIHC) was used by Steele, et al., to uncover a network of immune-suppressive cellular interactions [126]. This network was extensively heterogeneous, which suggests that a personalized approach should be used to tackle this disease by immunotherapy. Similar to the immune infiltration, several authors have shown distinct populations of CAFs in PDAC [127,128]. Using a scRNASeq-approach Dominguez, et al., were able to find a TGFβ-driven, LRRC15+ CAF lineage associated with poor outcomes in immunotherapy trial data [129]. Chan-Seng-Yue et al. utilized a single-cell multimodal approach that combined non-negative matrix factorization with sc-rnaseq analysis to expand the current understanding of pancreatic ductal adenocarcinoma (pdac) molecular subtypes. the study successfully identified five molecular subtypes ranging from classical-like and basal-like subtypes to “classical-like a”, “classical-like b”, “basal-like a”, “basal-like b”, and “hybrid” subtypes based on the presence of multiple expression signatures. notable findings from the study include the coexistence of classical-like and basal-like subtypes within the same tumor and a positive correlation between basal-like and epithelial-to-mesenchymal transition (EMT) programs, which leads to poor prognosis. additionally, the study found evidence that individual cells in a tumor can exhibit both basal-like and classical-like subtype features, highlighting the high intra-tumoral complexity and heterogeneity present in PDAC tumors. Previously, two separate subpopulations of cancer-associated fibroblasts (CAFs) had been identified, known as myofibroblastic CAFs (MyoCAFs) and inflammatory CAFs (iCAFs). MyoCAFs highly express α-smooth muscle actin (αSMA) and fibroblast activation protein (FAP). They are found in close proximity to neoplastic cells, forming a periglandular ring that surrounds tumor cell clusters. Meanwhile, iCAFs are more heterogeneously distributed and characterized by their ability to secrete pro-inflammatory cytokines, particularly Interleukin-6 (IL-6). These cells exhibit a high expression of IL-6 and low expression of αSMA protein, thus differentiating them from the previously mentioned MyoCAFs. These CAFs are called inflammatory CAFs due to their cytokine-secretory phenotype [130]. However, it became apparent that the two previously identified subpopulations of cancer-associated fibroblasts (CAFs) in PDAC tumors, iCAFs, and MyoCAFs, were not the only ones present. Hence, single-cell RNA sequencing (sc-RNAseq) was performed on samples from PDAC patients, along with adjacent normal tissue. this study confirmed the presence of iCAFs and MyoCAFs, along with their respective gene-expression profiles, while also adding information regarding different genes expressed in each population. revealing a novel caf subtype characterized by a high expression of major histocompatibility complex II (MHC-II) genes, which had previously only been associated with immune cells. Importantly, this study highlighted that this new subtype of CAF also expresses common fibroblast markers at similar levels to the previously mentioned subpopulations, confirming their identity as genuine fibroblasts. These findings indicate that this new subpopulation is capable of presenting antigens to CD4+ T cells [127]. These groundbreaking findings in the field of the tumor microenvironment (TME) in pancreatic ductal adenocarcinoma (PDAC) would not have been possible without the implementation of spatial and multimodal sc-RNAseq techniques. The spatial aspect of sc-RNAseq allows for the analysis of gene expression within the context of the tumor architecture, providing valuable insights into the localization and interaction of different cell populations. Additionally, the multimodal approach enables the simultaneous detection of multiple molecular features, such as protein expression or cytokine secretion alongside transcriptomic profiling. This integration of different modalities enhances our understanding of the complex cellular dynamics and functional heterogeneity within the TME. Thus, the application of sc-RNAseq in spatial and multimodal contexts is a useful toolkit to unravel the intricate landscape of PDAC and shed light on the diverse subpopulations and interactions within the TME.

By utilizing single nuclear RNA sequencing (sn-RNAseq) technologies, alongside spatial and multimodal approaches such as CyTOF and mfiHC, researchers have been able to investigate complex cell-cell communication networks and immune cell signaling hubs. These techniques can serve to identify the feedback crosstalk between different cell types within the tumor microenvironment. Interestingly, some of the strongest interactions identified were between epithelial cells and immune cells, which may contribute to the development of an immunosuppressive environment in PDAC [131,132]. It is important to note that another recent study was able to detect changes in the tumor microenvironment (TME) of pancreatic ductal adenocarcinoma after chemotherapeutic treatment, revealing a more immunosuppressive environment. Finally, it is noteworthy that, using single-nuclear RNA sequencing (sn-RNAseq), researchers were able to detect the different subpopulations of cancer-associated fibroblasts (CAFs), including myofibroblastic cafs (myoCAFs), inflammatory cafs (iCAFs), and antigen-presenting cafs (apCAFs) [133].

### Exosomes in PDAC TME

Some of the main biomolecules found in exosomes in PDAC are nucleic acids such as DNA, RNA, microRNAs, long non-coding RNAs (lncRNA), or circular RNAs (circRNAs) [134,135]. M2 macrophages appear to be one of the key players in exosome-mediated communication in the PDAC TME. Several studies have shown their important role in the development of some of the most important clinical features. Exosomal cargo derived from M2 macrophages can influence resistance against one of the main lines of defense in PDAC, such as [136]. In addition, the exosomal content of this type of macrophage (mainly lncRNAs and miRNAs) stimulates the angiogenic process [104] and invasion and metastasis processes. This is achieved by various miRNAs contained in exosomes derived from M2 macrophages that favor these processes by stimulating the production of matrix metalloproteinases 9 (MMP9) [137], or altering the TGF-ß signaling pathway, favoring invasion and metastasis to the liver and lungs [138]. PDAC tumor cells can also secrete exosomes to stimulate distant hepatic stellate cells (HSCs) to secrete fibronectin, causing fibrosis in the liver and establishing a pre-metastatic niche to facilitate invasion of pancreatic tumor cells [139]. The escape from immunological surveillance is another aspect in which exosomes derived from malignant PDAC cells have an influence. It has been shown that such exosomes contain miRNAs or lncRNAs that alter antigen-presenting professional cells, such as dendritic cells, inhibiting expression of the major histocompatibility complex II (MHC II) and suppressing CD4+ T lymphocytes [140,141,142]. Pancreatic stellate cells can also be an important source of exosomes in PDAC, whose content is mainly based on miRNAs. These favor tumor progression, since some of their main targets are tumor suppressor genes such as PTEN [143] Similarly, they induce migration and EMT [144]. Communication between tumor cells and CAFs can also be mediated by exosomes, which can cause metabolic reprogramming in CAFs, from oxidative phosphorylation to aerobic glycolysis. This reprogramming produces metabolic intermediates such as lactic acid or ketone bodies, which are subsequently ingested by tumor cells to promote growth and tumor progression [145] These metabolic products can also be encapsulated in exosomes and transported to other tumor cells, which ingest them and can use them in carbon metabolism, favoring growth and tumor progression under conditions of low nutrients or stress [146].

## 8. Therapeutic Approaches against PDAC

As mentioned, PDAC is a disease that presents a very dense stroma, often representing up to 90% of the tumoral mass that originates from tumor-associated fibroblasts (CAFs). This stroma is composed of two main different types: “normal” and “activated”. This is particularly relevant due to the relationship between these two states and prognosis since the activated stroma is associated with a higher malignancy and worst prognosis [147]. Similarly, there are also different PDAC molecular subtypes, as assessed by whole-genome RNA expression studies [125,148,149]. Even when different authors have described different molecular subtypes, these share many characteristics, such as the classical/canonical subtypes which express genes related to a more epithelial phenotype and the quasi-mesenchymal/basal-like subtype which expresses genes associated with a mesenchymal phenotype and behaves more aggressively. It is also important to highlight that the latter overlap with other basal-like tumors such as basal-type breast tumors. The stromal and PDAC subtypes could be used in the future to give patients a more personalized treatment scheme [125,147,149]. Adding to the complexity, previous studies showed that both classical and basal-like can coexist in the same tumor, within the same patient, or even in ex-vivo cultures. This increases the difficulty of the diagnosis and treatment of patients [150]. 

Perhaps the most important PDAC prognosis factor is the time of detection. Surgery followed by chemotherapy provides the only possible cure so far. However, only 10–20% of patients have this possibility since the remaining 80–90% present with locally advanced disease, non-resectable tumor, or metastasis [151,152]. In PDAC, the main adjuvant chemotherapeutic agents used in its treatment are nucleoside analogs, such as gemcitabine, and the pyrimidine analog 5-Fluorouracil (5-FU), usually used individually or in combination with other methods, such as radiotherapy (RT) [153,154]. Combination therapy is the gold standard, as it has been demonstrated to double the patient’s survival; FOLFIRINOX is a compound of folinic acid, 5-FU, irinotecan, and oxaliplatin [155]. CSCs could be involved in the primary and secondary resistance in PDAC patients [156]. CSC resistance is due to several mechanisms, including the overexpression of ABCG family transporters, as they are able to efflux drugs efficiently, as demonstrated by experimental approaches using a fluorescent dye (Rhodamine 123 or Hoescht 33343) [157]. The ABC transporters can act upon an extensive range of toxic agents. Therefore, chemotherapy is moderately effective to eliminate bulk tumor cells, whereas CSCs survive [158]. To address this problem, some authors have proposed the use of drug combinations to eliminate CSCs, such as gemcitabine in combination with salinomycin to eliminate pancreatic stem cell numbers [159]. An additional example was the of a kinase 1 (*Chk1*) inhibitor on CSCs to make them susceptible to the effects of gemcitabine treatment [160]. 

Radiotherapy has been shown to improve PDAC resectability in locally advanced or primarily inoperable/borderline-operable patients [161,162]. Previous studies have shown that CSCs are radioresistant and responsible for the relapse of many cancer types, including PDAC. There are several ways in which CSCs evade radiotherapy. Hypoxic TME helps CSCs become highly radioresistant and in conjunction with factors controlling angiogenesis such as vascular endothelial growth factor (*VEGF*), lack of nutrients, and an acidic environment. There are also intrinsic factors contributing to CSCs being radio-resistance, such as efficient DNA repair mechanisms, high levels of free radical scavenging molecules, and stimulated DNA checkpoints [159,163,164,165,166]. Since these tumors often present radio-resistance, radio-sensitization using chemotherapeutic agents (Gemcitabine, capecitabine, nab-paclitaxel, 5-FU, etc.) is commonly used in order to improve radiotherapy effectiveness. In addition, several authors have explored new ways to decrease radio-resistance in CSC. For example, it has been shown that the population of CD133+ cells is enriched after RT treatment; this may be due to the DNA repair ability present in CSCs through activation of kinase checkpoint 1 (*Chk1*) and *Chk2*. Conceivable, exposure of these cells to Chk inhibitors could relieve this checkpoint to improve the response to radiation [158,163].

Immunotherapy has revolutionized oncology in recent years. Even when spectacular particular responses have been observed, not all patients benefit from them [167]. While immunotherapy has become an important second-line or even first-line treatment for some cancer types, such as melanoma or lung adenocarcinoma, it has not been shown to be effective in gastrointestinal cancer types such as PDAC. The main immunotherapy drugs used to act upon the PD-1/PD-L1 pathway are useful in tumors with high mutational load, microsatellite instability, or mismatch repair deficiency. Unfortunately, only 1–2% PDAC patients have these characteristics [168]. Adding to this, patients with these tumors have a highly immunosuppressive TME and a dense stroma which is not only a physical barrier to the infiltration of immune cells but also contributes to an inadequate immunotherapy response [169]. Several researchers are working on creating vaccines against tumor-associated antigens (TAAs) to teach the immune system to recognize tumor cells expressing TAAs more quickly and generate a stronger immune response. However, there are limitations such as the low number of T cells in the tumor microenvironment (TME) in pancreatic cancer, the dense stroma mentioned earlier, and the presence of immune suppressive cells in the TME [170]. Another limitation with respect to vaccination is that the CSC number within a tumor typically is just 1–2%. For this reason, a recent work used a broad-spectrum vaccination approach designed to target all cells expressing the α-gal epitope. This strategy would include both non-CSCs and CSCs. As expected, there was a strong immune response and activation of different immune cells, mainly T regulatory cells. It also led to the production of a large number of antibodies against several tumor-associated antigens (TAAs) with the same effect demonstrated on the pancreatic cancer stem cell population CD44+CD24+ [171]. Although the effects were not seen on the pancreatic cancer stem cells with the most tumorigenic potential, this is a promising result. 

The quiescence in CSCs presents a significant obstacle to effectively targeting this population. As these cells are believed to be arrested in the G0 cell cycle phase, it becomes difficult to target high-proliferating cells with drugs. Blocking their self-renewal and differentiation capabilities may be the only effective way of killing the CSCs. Additionally, the “stem cell niche” is an important therapeutic feature in CSCs. This highly specific microenvironment provides the necessary characteristics and protection to maintain these cells while also playing a key role in metastasis, invasion, and tumor progression according to some authors [172].

In PDAC, the genes responsible for both initiation and progression are well documented since they are present in most PDAC cases (*KRAS*, *TP53*, *SMAD4*, *CDKN2A*) [173,174,175,176,177]. This homogeneous genetic origin seems odd with the heterogeneous nature of this disease. This may be due to an epigenetic rather than a genetic component, as has been seen in some studies [178]. This could be beneficial for the patients since there are new drugs able to regulate the epigenome [178]. Support for this comes from a recent study conducted to answer the question of why people might develop pancreatic cancer up to 12 years after having an episode of pancreatitis [179]. In this study, the authors found that even when the pancreatic tissue returns to normality after the inflammatory event, a small number of cells present a persistent alteration of the epigenome, as assessed by the presence of differentially accessible chromatin zones, which favors the latter development of PDAC. This clearly opens the door for the potential use of epigenome-acing drugs for the treatment of this cancer type [179].

Several studies have attempted to eliminate or sensitize pancreatic CSCs through the use of inhibitors, including ROS inhibitors. Combinations of therapeutic regimens blocking the JNK-ROS axis have shown promising outcomes. Other inhibitors, including mTOR inhibitors, glucose transporter 1 (GLUT1) inhibitors, histone deacetylase (HDAC) inhibitors, and drugs like salinomycin or sorafenib (an FDA-approved drug) that target pancreatic cancer stem cells, have also shown promise. Combinations of different drugs, such as gemcitabine plus irinotecan, have effectively eliminated the CSCs CD24+CD44+ population. However, every approach is specific to the tumor or patient, which is likely due to the high complexity and heterogeneity present in PDACå [172].

## 9. Looking for a Better CSC Characterization and Isolating System

As previously mentioned, there are many different markers used to isolate pancreatic cancer stem cells (CSCs), with each population exhibiting the necessary traits to qualify as CSCs. Nevertheless, a comprehensive study is still needed to determine the most effective combination of cell surface markers that accurately define stemness in pancreatic ductal adenocarcinoma (PDAC). Addressing this discrepancy is crucial for identifying the best therapeutic targets and biomarkers that can be utilized for detection and prognosis. To this end, Tang et al. devised a lentiviral-based system to functionally detect stem cells. The researchers employed the triad of master transcriptional regulators, namely, *SOX2*, *OCT4*, and *NANOG*, as a reporter system to sustain the embryonic-cell phenotype. Knowing that the pluripotency in differentiated cells could only be induced by a combination of three transcription factors, the team hypothesized that *SOX2* and *OCT4* must be activated in tumor stem cells. Consequently, they created a plasmid with a promoter for both and associated it with the reporter gene GFP for identifying and characterizing cells with active factors. GFP+ cells isolated by FACS exhibited CSC functionality, manifested via asymmetric divisions, self-renewal, therapy resistance, spheroid formation, zero expression of differentiation markers, and the creation and sustenance of new tumors in vivo. As a result, such a system appears highly promising for studying this specific cell subtype in different cancer types [180].

## 10. Conclusions

The tumor microenvironment is a key player in PDAC progression since it provides a complex network of different cell types, molecules, and extracellular matrix components that interact with cancer cells. The highly complex tumor microenvironment is largely responsible for intra- and inter-tumoral heterogeneity in PDAC, which in turn influences response to therapy. A deep understanding of TME should help to design new therapeutic approaches. In addition, efforts to establish a global agreement on molecular subtypes, and agents to sensitize CSCs to different therapeutic schemes can improve prognosis and treatment of this challenging disease.

## Data Availability

Not applicable.
